# Estudio de verificación de precisión y veracidad en un sistema Atellica

**DOI:** 10.1515/almed-2020-0041

**Published:** 2020-09-04

**Authors:** Alberto Vílchez Rodríguez, Julia González Cantó, Sara Esteve Poblador, Carmen Valldecabres Ortiz, Pedro L. Estela Burriel

**Affiliations:** Área de Diagnóstico Biológico, Hospital Universitario La Ribera, Alzira, Valencia, España

**Keywords:** bioquímica, comparación de métodos, evaluación de métodos, inmunoquímica

## Abstract

**Objetivos:**

El laboratorio clínico debe usar solamente procedimientos de medida validados. La precisión es una de las características más importantes para seleccionar, incorporar, validar y verificar un procedimiento de medida. El objetivo fue verificar la precisión y veracidad de varias magnitudes en distintos analizadores de bioquímica e inmunoquímica.

**Materiales:**

Los analizadores en uso fueron Advia 1800^®^, Immulite^®^2000 y CentaurXP^®^, y el sistema evaluado fue Atellica^®^Solution. Se evaluaron cinco magnitudes para bioquímica y cinco para inmunoquímica. Para estudiar la imprecisión se utilizaron materiales de control comercial de BioRad^®^ y Siemens^®^, calculando la imprecisión intraserial e interserial analizando tres replicados de cada control en una única serie cada día, durante 5 días. Para el estudio de veracidad se analizaron 40 muestras de suero diferentes mediante el análisis de diferencias y regresión lineal.

**Resultados:**

Los valores obtenidos para la imprecisión intraserial e interserial fueron menores a los proporcionados por el fabricante en todas las magnitudes estudiadas. En el estudio de veracidad, existe error sistemático proporcional pero no constante para algunas magnitudes.

**Conclusiones:**

Según los coeficientes de variación (CV%) obtenidos en el Atellica^®^Solution se verificaron las especificaciones de imprecisión proporcionadas por el fabricante y las establecidas por el laboratorio. Respecto al estudio de veracidad sería necesario para algunos parámetros, revisar las condiciones de calibración y ampliar el rango de muestras estudiado.

## Introducción

Para una correcta interpretación clínica y poder comparar distintos resultados de un paciente, estos deben ser exactos.

La norma ISO 15189 para la acreditación de los laboratorios clínicos establece que el laboratorio debe utilizar solamente procedimientos validados para confirmar que son adecuados para la utilización prevista, siendo la precisión una de las características metrológicas más importantes a tener en cuenta para la selección, implantación y validación de un procedimiento de medida [1].

Los objetivos del estudio son, por una parte, verificar las prestaciones de precisión proporcionadas por el fabricante en condiciones de repetibilidad y en condiciones intermedias, calculando la imprecisión intraserial e interserial. Por otra parte, verificar la veracidad (error sistemático [ES]) de un procedimiento de medida nuevo mediante la comparación con el procedimiento de medida en uso, y así comprobar la intercambiabilidad de ambos procedimientos permitiendo mantener los valores de referencia asignados a cada magnitud evaluada.

## Materiales y métodos

Este estudio se realizó en el Área de Diagnóstico Biológico del Hospital Universitario La Ribera (Alzira).

Los analizadores de bioquímica en uso fueron de Siemens Healthineers: Advia 1800^®^ para bioquímica clínica, e Immulite^®^2000 y ADVIA CentaurXP^®^ para inmunoquímica. El sistema evaluado fue Atellica^®^Solution (bioquímica e inmunoquímica), asumiendo como valor verdadero el que proporcionan los procedimientos de medida en uso.

Las magnitudes estudiadas en el Advia 1800^®^ fueron: Srm-calcio; c.sust., Srm-L-lactato deshidrogenasa; c.cat. (LDH), Srm-ferritina; c.sust., Uri-albúmina; c.masa. y Uri-proteína total; c.masa.; en el ADVIA CentaurXP^®^: Srm-25-OH-colecalciferol; c.sust. (vitamina D) y Uri-cortisol; c.sust.; y en el Immulite^®^2000: Srm-N-terminal proBNP; c.sust. (NT-proBNP), Srm-proteína A plasmática asociada al embarazo; c.arb. (PAPP-A) y Srm-gonadotropina coriónica humana subunidad β; c.arb. (β-HCG).

El método de los procedimientos de medida en uso y procedimientos de medida evaluados para bioquímica fue la espectrofotometría, con cambios únicamente en la reacción para la LDH (para el método en uso de piruvato a L-lactato y para el método evaluado de L-lactato a piruvato). El método para las determinaciones de inmunoquímica, tanto en el procedimiento de medida en uso como el procedimiento de medida evaluado fue quimioluminiscencia. En el caso de la ferritina se cambió de un método inmunoturbidimétrico para el procedimiento de medida en uso a quimioluminiscencia directa para el procedimiento de medida evaluado.

Se utilizaron materiales de control comercial de BioRad^®^: Multiqual (calcio, ferritina y LDH), Liquichek Cardiac Markers (NT-proBNP), Immunoassay plus control (vitamina D) y Urine Chemistry (proteína total, albúmina, y cortisol en orina), y control comercial de Siemens^®^: IMMULITE^®^Systems PAA (PAPP-A) e IMMULITE^®^Systems FBC (β-HCG). Se consideraron para cada magnitud dos niveles de control, uno con valor cercano al de decisión clínica y otro con valor patológico, siendo estos valores similares a los utilizados por el fabricante en su estudio de imprecisión.

Para estimar la imprecisión, se siguió el documento de la SEQC basado en las guías publicadas por el Clinical and Laboratory Standards Institute (CLSI) [2], [3]:

– Se analizaron tres replicados de cada control en una única serie cada día, durante 5 días.
– Se calculó la imprecisión intraserial (*CV*_*r*_):

$$\begin{array}{l} s_{r}=\sqrt{\frac{\sum_{d=1}^{D}\sum_{i=1}^{n}{\left(x_{di}-X_{d}\right)}^{2}}{D\left(n-1\right)}}\\ {CV}_{r}=100\cdot \frac{S_{r}}{X_{t}} \end{array}$$

Siendo *S*_*r*_ = desviación estándar intraserial, *X*_*di*_ = resultado del replicado *i* en el día *d*, *X*_*d*_ = media de resultados del día *d*, *D* = número de días (cinco), *n* = número de replicados por día (tres), y *X*_*t*_ = media de todos los resultados.

– Se calculó la imprecisión interserial (*CV*_*T*_):

$$\begin{array}{c} s_{b}^{2}=\frac{\sum_{i=1}^{D}{\left(X_{d}-X_{t}\right)}^{2}}{D-1}\\ s_{T}=\sqrt{\frac{n-1}{n}S_{r}^{2}+S_{b}^{2}} \end{array}$$

$$CV_{T}=100\cdot \frac{S_{T}}{X_{t}}$$

Siendo *S*_*b*_ = desviación estándar interdiaria, *X*_*d*_ = media de resultados del día *d*, *X*_*t*_ = media de todos los resultados, *D* = número de días (cinco), *S*_*T*_ = desviación estándar interserial, *S*_*r*_ = desviación estándar intraserial y *n* = número de replicados por día (tres).

– Se compararon los valores obtenidos de imprecisión intraserial e interserial con las especificaciones proporcionadas por el fabricante para el mismo rango de concentraciones. Los valores estimados debían ser iguales o inferiores a los especificados por el fabricante.

Para el estudio de veracidad se analizaron 40 muestras de diferentes pacientes en 5 series analíticas por los dos procedimientos de medida que, tras eliminar los valores aberrantes, se redujeron a 38 muestras para cortisol y β-HCG. Aproximadamente, el 50% de las muestras procesadas para cada magnitud tenían valores fuera del intervalo de referencia y todas las magnitudes presentaban concentraciones distribuidas uniformemente a lo largo del intervalo de medida.

Para el análisis de los resultados del estudio de veracidad se utilizaron dos métodos que proporcionan información complementaria [4]:

– Análisis de las diferencias: se calcularon las diferencias entre el resultado obtenido con el procedimiento evaluado (x) y el resultado con el procedimiento de comparación (y), se calculó el promedio de cada pareja de resultados y las diferencias relativas porcentuales (*DR*). La diferencia entre los resultados de los dos procedimientos se describió mediante el valor de la media de las diferencias absolutas (*Dm*) o relativas (*DRm*), calculándose los intervalos de confianza del 95% (IC 95%).
– Regresión lineal: se representaron los valores de “y” frente a “x”, se obtuvieron los valores de la pendiente (b) y la ordenada en el origen (a), con sus respectivos intervalos de confianza del 95%.

## Resultados

En la Tabla 1 se resumen los valores obtenidos en el estudio de imprecisión intraserial (*CV*_*r*_) e interserial (*CV*_*T*_) junto a los valores declarados por el fabricante.

**Tabla 1:** Resultados de imprecisión (%).

Magnitud	Control	Media $\bar{x}$	Imprecisión, %			
			*CV_r_* Fabricante	*CV_r_* Estimado	*CV*_*T*_ Fabricante	*CV*_*T*_ Estimado
Srm-calcio; c.sust.	Nivel 1	1,54 mmol/L	1,2	0,7	1,7	0,7
	Nivel 3	3,27 mmol/L	0,6	0,5	0,7	0,7
Srm-L-lactato deshidrogenasa; c.cat.	Nivel 1	189 U/L	0,9	0,7	1,0	0,9
	Nivel 3	403 U/L	0,7	0,4	1,0	0,6
Srm-ferritina; c.sust.	Nivel 1	92,1 pmol/L	1,4	1,4	4,2	1,4
	Nivel 3	143,8 pmol/L	1,3	1,1	4,4	1,0
Srm-propéptido natriurético cerebral N-terminal; c.sust.	Nivel 1	15,4 pmol/L	2,3	1,8	3,9	3,7
	Nivel 3	377,8 pmol/L	1,8	1,7	3,7	2,8
Uri-albúmina; c.masa.	Nivel 1	292 mg/L	1,4	1,0	3,6	0,9
	Nivel 3	409 mg/L	1,2	0,6	1,4	0,7
Uri-proteína total; c.masa.	Nivel 1	121 mg/L	4,9	1,6	5,3	1,8
	Nivel 3	549 mg/L	1,2	0,7	1,9	0,6
Srm-25-OH-colecalciferol; c.sust.	Nivel 1	60,3 nmol/L	5,0	4,8	8,0	4,3
	Nivel 3	226,9 nmol/L	1,8	1,6	3,2	2,5
Uri-cortisol; c.sust.	Nivel 1	19,5 nmol/día	2,6	2,3	8,9	4,1
	Nivel 3	63,7 nmol/día	5,5	4,7	9,6	7,9
Srm-proteína A plasmática asociada al embarazo; c.arb.	Nivel 1	1,54 UI/L	2,7	2,2	3,4	2,3
	Nivel 3	4,17 UI/L	2,9	2,2	4,1	3,6
Srm-gonadotropina coriónica humana subunidad β; c.arb.	Nivel 1	20,03 UI/L	1,5	1,1	2,5	2,4
	Nivel 3	177,7 UI/L	1,3	1,0	2,3	2,0

*CV_r_*, coeficiente de variación intraserial; *CV_T_*, coeficiente de variación interserial.

En la Tabla 2 se resumen los datos del estudio de veracidad, para el análisis de diferencias y regresión lineal.

**Tabla 2:** Resultados estudio de veracidad.

		Suero							Orina		
		Srm-calcio	Srm-L-lactato deshidrogenasa	Srm-ferritina	Srm-propéptido natriurético cerebral N-terminal	Srm- proteína A plasmática asociada al embarazo	Srm-gonadotropina coriónica humana subunidad β	Srm-25-OH-colecalciferol	Uri-cortisol	Uri-albúmina	Uri-proteína total
Análisis de diferencias	IC 95% *Dm*	(−0,03; 0,13)	(200,17; 253,34)	(−27,29; −4,10)	(−74,73; 599,06)	(−1,68; −0,98)	(6,23; 9,20)	(−0,77; 1,34)	(−0,3; 6,14)	(−34,84; 130,39)	(−2,91; 6,88)
	IC 95% *DRm*	(−0,25; 1,35)	(73,19; 78,69)	(−1,15; 3,22)	(4,68; 16,11)	(−30,51; −24,81)	(16,29; 21,69)	(−2,7; 7,62)	(−6,18; 20,48)	(−2,59; 16,97)	(−3,82; 19,08)
Regresión lineal	IC 95% *a*	(−1,02; 0,87)	(−3,29; 77,06)	(−2,31; 17,85)	(−370,20; 116,17)	(−0,48; 0,33)	(−2,75; 3,62)	(0,61; 5,16)	(−10,14; −2,14)	(−30,25; 3,76)	(−4,24; 0,63)
	IC 95% *b*	(0,91; 1,12)	(1,81; 2,22)	(0,92; 0,96)	(1,25; 1,43)	(0,71; 0,83)	(1,12; 1,29)	(0,78; 0,9)	(1,43; 1,91)	(1,13; 1,15)	(1,05; 1,07)
	*r*	0,898	0,902	0,997	0,955	0,945	0,956	0,899	0,848	0,999	0,999

IC 95%, intervalo de confianza 95%; *Dm*, diferencias absolutas; *DRm*, diferencias relativas; *a*, valores de la ordenada en el origen; *b*, valores de la pendiente; *r*, coeficiente de correlación.

Según el análisis de diferencias:

– No existe ES constante ni proporcional para proteína total, albúmina y cortisol en orina; y calcio y vitamina D en suero, ya que el IC 95% de *Dm* y *DRm* incluyen el valor 0.
– No existe ES constante (IC 95% *Dm* incluye el 0), pero sí existe ES proporcional (IC 95% *DRm* no incluye el 0) para NT-proBNP en suero.
– Existe ES constante (IC 95% *Dm* no incluye el 0), pero no existe ES proporcional (IC 95% *DRm* incluye el 0) para ferritina en suero.
– Existe ES constante (IC 95% *Dm* no incluye el 0), y ES proporcional (IC 95% *DRm* no incluye el 0) para LDH, PAPP-A y β-HCG en suero.

Según el análisis de regresión lineal:

– No existe ES constante ni proporcional para calcio en suero, ya que el IC 95% de la ordenada en el origen contiene el valor 0 y el IC 95% de la pendiente contiene el valor 1.
– No existe ES constante (IC 95% ordenada origen contiene el 0), pero sí existe ES proporcional (IC 95% pendiente no contiene el 1) para proteína total y albúmina en orina; y PAPP-A, β-HCG, NT-proBNP, ferritina y LDH en suero.
– Existe ES constante (IC 95% ordenada origen no contiene el 0), y ES proporcional (IC 95% pendiente no contiene el 1) para cortisol en orina, y vitamina D en suero.

**Figura 1:**
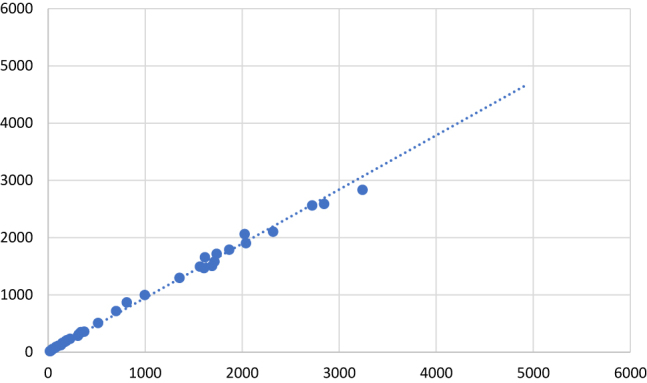
Estudio de regresión lineal para ferritina en suero (pmol/L).

En la Figura 1, aparece la gráfica del estudio de regresión lineal para la ferritina en suero.

## Discusión

El estudio de precisión se realizó bajo condiciones de repetibilidad e intermedias, siendo éstas últimas las que tienen mayor interés en el laboratorio clínico. Según indican los coeficientes de variación (CV%) obtenidos en el Atellica^®^Solution, se verifican las especificaciones de imprecisión para los distintos procedimientos de medida proporcionados por el fabricante para todas las magnitudes estudiadas, y además se cumplen las especificaciones de calidad analítica establecidas por el laboratorio basadas en la variabilidad biologica [5] para calcio, LDH, ferritina, albúmina, proteína total y NT-proBNP. Para la vitamina D, PAPP-A, cortisol y β-HCG no existen datos de variabilidad biológica pero cumplen los requisitos basados en el estado del arte obtenidos a través del programa externo de calidad de nuestro laboratorio.

En cuanto al estudio de veracidad, realizado mediante el análisis de las diferencias y el análisis de regresión lineal, se confirma que no existe ES significativo constante ni proporcional para proteína total, albúmina y cortisol en orina y ferritina, calcio y vitamina D en suero. Sin embargo, no existe ES constante, pero si proporcional para NT-proBNP, LDH, β-HCG y PAPP-A en suero. Para NT-proBNP, LDH y β-HCG la pendiente es mayor a 1, por tanto, los resultados del procedimiento de medida nuevo pueden ser proporcionalmente más altos respecto a los del procedimiento de comparación. Para PAPP-A, la pendiente es menor a 1 pudiéndose obtener resultados más bajos en el procedimiento de medida nuevo.

Según los coeficientes de correlación (r) obtenidos por el análisis de regresión lineal, son inferiores a 0,975 para NT-proBNP, LDH, PAPP-A, β-HCG, vitamina D y calcio en suero y cortisol en orina, por tanto, el intervalo de valores debe ser ampliado con muestras adicionales.

Además para LDH, el procedimiento de medida nuevo es diferente al de uso por lo que las diferencias detectadas pueden deberse a que existe un ES derivado de la diferencia entre los dos procedimientos de medida comparados y sería conveniente aplicar diferentes valores de referencia.
